# Differential inhibitory action of apixaban on platelet and fibrin components of forming thrombi: Studies with circulating blood and in a platelet-based model of thrombin generation

**DOI:** 10.1371/journal.pone.0171486

**Published:** 2017-02-13

**Authors:** Lluis Pujadas-Mestres, Irene Lopez-Vilchez, Eduardo Arellano-Rodrigo, Joan Carles Reverter, Antonio Lopez-Farre, Maribel Diaz-Ricart, Juan Jose Badimon, Gines Escolar

**Affiliations:** 1 Department of Hemotherapy and Hemostasis, Hospital Clinic of Barcelona, Centre of Biomedical Diagnosis (CDB), Institute of Biomedical Research August Pi i Sunyer (IDIBAPS), University of Barcelona, Barcelona, Spain; 2 Department of Medicine, School of Medicine, Complutense University, Madrid, Spain; 3 Atherothrombosis Research Unit, Cardiovascular Institute, Mount Sinai School of Medicine, New York, New York, United States of America; Monash University, AUSTRALIA

## Abstract

**Introduction:**

Mechanisms of action of direct oral anticoagulants (DOAC) suggest a potential therapeutic use in the prevention of thrombotic complications in arterial territories. However, effects of DOACs on platelet activation and aggregation have not been explored in detail. We have investigated the effects of apixaban on platelet and fibrin components of thrombus formation under static and flow conditions.

**Methods:**

We assessed the effects of apixaban (10, 40 and 160 ng/mL) on: 1) platelet deposition and fibrin formation onto a thrombogenic surface, with blood circulating at arterial shear-rates; 2) viscoelastic properties of forming clots, and 3) thrombin generation in a cell-model of coagulation primed by platelets.

**Results:**

In studies with flowing blood, only the highest concentration of apixaban, equivalent to the therapeutic C_max_, was capable to significantly reduce thrombus formation, fibrin association and platelet-aggregate formation. Apixaban significantly prolonged thromboelastometry parameters, but did not affect clot firmness. Interestingly, results in a platelet-based model of thrombin generation under more static conditions, revealed a dose dependent persistent inhibitory action by apixaban, with concentrations 4 to 16 times below the therapeutic C_max_ significantly prolonging kinetic parameters and reducing the total amount of thrombin generated.

**Conclusions:**

Our studies demonstrate the critical impact of rheological conditions on the antithrombotic effects of apixaban. Studies under flow conditions combined with modified thrombin generation assays could help discriminating concentrations of apixaban that prevent excessive platelet accumulation, from those that deeply impair fibrin formation and may unnecessarily compromise hemostasis.

## Introduction

Platelets contribute to the precipitating events leading to acute coronary occlusion [1]. Plaque disruption facilitates the interaction of flowing blood with the inner components of atherosclerotic lesions, collagen and tissue factor among them; leading to local thrombin generation and acute thrombus formation [2]. Mechanisms by which plaque rupture leads to occlusive or non-occlusive thrombi are complex and probably potentiated by the presence and/or transport of hyper-reactive platelets and soluble agonists, such as thrombin towards the injured vessel [3]. Occlusive events in arterial vessels occur under a wide range of hemodynamic conditions, ranging from extreme to depressed flows, including situations with turbulences or stasis in pathological stenotic vessels.

It has been proposed that exposure of vascular tissue factor (TF) at sites of disrupted atherosclerotic plaques plays a critical role in the local generation of thrombin favoring the growth of occlusive thrombi. Assembly of the TF-FVIIa complex on the anionic phospholipids expressed at the activated cell-membranes is important for optimal thrombin generation and blood coagulation. Actual models of coagulation contemplate the implication of cellular and enzymatic mechanisms in three differentiated steps: initial activation, propagation and thrombin generation [4,5,6]. Although the more recent cell-based model of coagulation does not take into account the elevated shear rate conditions usually involved in the onset of acute coronary events, the concepts may still apply to post-stenotic arterial territories or during the consolidation of occlusive thrombi characterized by slow flow or even almost static conditions.

Blood flow conditions, and more specifically blood shear rates, are essential in the pathophysiology of venous and arterial thrombotic complications [7]. Interaction of platelets with a damaged vessel wall and thrombus formation are shear-dependent processes [8]. Besides, rheological properties of blood locate circulating platelets close to the boundary layer, where they recognize and interact with elements of the subendothelium exposed upon vessel damage. Rupture of an atherosclerotic plaque, will expose tissue factor and collagen to the bloodstream and to platelets, triggering the formation of a thrombus and subsequent ischemic complications of acute coronary syndromes (ACS) [9,10]. The size, stability and composition of this thrombus will determine the severity of the ACS [11,12].

Thrombi generated on a ruptured plaque contain platelets and fibrin [12]. The initial activation and aggregation of platelets are important processes in thrombus formation; however, its stability is dependent on their fibrin content and polymerization. Disrupted atherosclerotic lesions are rheologically characterized by increasing shear rates reaching maximal levels at the apex of the stenosis. These high shear rate conditions are magnified by the occlusive nature of the forming thrombus on the disrupted lesion. Distal to the apex, the local shear rate progressively decelerates and even switches to negative values due to distal post-thrombus turbulence [13–15]. Reduced flow conditions at the proximal and distal portions of the growing thrombi favor local thrombin generation, therefore increasing the stability of the thrombi by incorporating additional platelets into a polymerizing fibrin mesh.

Antiplatelet agents and anticoagulants therapies such as heparins have proven clinically successful, though a significant number of recurrent events may still occur. Direct oral anticoagulants (DOACs) can be considered equally effective than warfarin, but safer and easier to use. Despite these advantages, there is limited information on the effects of DOACs in the ACS setting; and the possible synergism of a combined antiplatelet plus anticoagulant therapy has not been investigated in detail [16]. The well-established inhibitory activity of DOACs on thrombin generation should be considered a major asset; however, their effect on platelet-mediated procoagulant activity has not been explored yet. In fact, some clinical studies have shown contradictory results depending on the DOAC and doses investigated [17].

In summary, platelet activation, local thrombin generation and fibrin formation are three key elements contributing to the formation of occlusive thrombi in arterial territories. The mechanisms of action of DOACs support their potential therapeutic use in the prevention of thrombotic complications in arterial vasculature however effects of these agents on platelet activation and aggregation have not been investigated in detail. In the present studies, we have applied experimental approaches under dynamic and steady flow conditions to investigate the effects of apixaban on different aspects of thrombus formation. More specifically, we assessed the inhibitory effects of apixaban on: 1) platelet deposition and fibrin formation onto a thrombogenic surface, in an experimental model of thrombosis with blood circulating at arterial shear-rates; 2) clot formation kinetics using thromboelastometry; and 3) thrombin generation applying a cell-based model of coagulation primed by platelets.

## Materials and methods

Our investigations conform with the Directive 2010/63/EU of the European Parliament on the protection of animals used for scientific purposes; and with the principles outlined in the Declaration of Helsinki. The study has been approved by the Hospital Clinic Ethical Committee of Clinical Investigation (CEIC registry: 2011/6837). Healthy donors provided an informed written consent before collection of blood samples. The protocol to isolate rabbit aortas, to be used as thrombogenic substrata, was also approved by the Animal Ethical Committee of the University of Barcelona (CEEA registry: 473/12). Rabbits were handled following the applicable regulations and guidelines of the Animal Experimental Ethics Committee of the University of Barcelona.

### Experimental design

The aim of the present study was to assess the effects of apixaban at therapeutic and subtherapeutic doses in different models of hemostasis: 1) Modifications in the adhesive-cohesive properties of platelets and in fibrin formation were evaluated with perfusion devices, using whole blood circulated through damaged vascular segments, and a shear rate similar to those found at medium-sized arteries; 2) changes in thromboelastometry parameters during clot formation of citrated blood; and 3) contribution of platelets to kinetics of local thrombin generation, using an *in house* cell-based model of thrombin generation primed by platelets, in a fluorimetric assay with a fluorogenic substrate (Technoclone GmBH, Austria).

### Reagents

Phosphate buffered saline (PBS) was from Gibco BRL Life Technologies (Paisley, UK). The embedding kit JB-4 for histological processing was from Polyscience (Warrington, USA). ROTEM Thromboelastometry reagents, ex-TEM^®^ and star-TEM^®^, were from Pentapharm GmbH (Munich, Germany). Thrombin generation was assessed with the fluorogenic substrate from Technoclone GmbH (Vienna, Austria). Tissue factor exposed on phospholipid vesicles to trigger thrombin generation was Thromborel^®^S, from Siemens Healthcare (Marburg, Germany).

### Apixaban

The apixaban principle was kindly provided by Bristol-Myers Squibb (NY, USA). Apixaban was initially dissolved in ethanol, and subsequently diluted in saline to a final stock concentration of 1 μg/mL. Blood samples where incubated with different concentrations of apixaban (0, 10, 40 and 160 ng/mL) for 30 min at 37°C. The 160 ng/mL concentration is equivalent to the C_max_ at steady state after a 5 mg twice daily dosage, recommended for the thromboprophylaxis in patients with atrial fibrillation [18]. The 40 ng/mL is compatible with the C_min_ after the same treatment regimen, and the 10 ng/mL would be compatible with the C_min_ achieved after a after a single administration of 2.5 mg apixaban [19].

### Blood collection and platelets isolation

Blood samples were drawn from healthy donors who in the previous 10 days had not taken any drug known to affect platelets or the coagulation system. Blood was collected into BD Vacutainer^™^ citrate tubes leading to a final citrate concentration of 11 mM. For thrombin generation studies, whole blood was separated into plasma and isolated platelets. Citrated plasma was obtained by centrifugation of tubes at 1100xg for 5 min. Platelets were separated as platelet-rich plasma (120xg, 7 min) and washed 3-times with a buffer containing 93 mM sodium citrate, 7 mM citric acid, 140 mM dextrose, 5 mM adenosine and 3 mM theophylline (pH 6.5). The final pellet was resuspended at 1x10^6^ platelets/μL in Hanks’ balanced salt solution (136.8 mM NaCl, 5.3 mM KCl, 0.6 mM Na_2_HPO_4_, 0.4 mM KH_2_PO_4_, 0.2 mM NaH_2_PO_4_-2H_2_O) supplemented with 2.7 mM dextrose and 4.1 mM NaHCO_3_ (pH 7.2). Platelets suspensions were allowed to rest at 37°C for 30 min [20].

### Perfusion studies

Whole blood aliquots incubated with apixaban were recalcified with a CaCl_2_ solution, and immediately perfused through annular chambers exposing a damaged vascular segment from rabbit aorta, as thrombogenic substrata [21].

### Preparation of thrombogenic surfaces

Aortas were extracted from young female New Zealand rabbits (2.8–3.0 Kg; http://www.granjasanbernardo.com/). Rabbits were sedated with a combination of intramuscular ketamine (30 mg/Kg) + xylazine (5 mg/Kg), and further euthanized using an overdose of anesthetic (intravenous sodium pentobarbital; 500 mg/Kg). Death was confirmed by total bleeding. Rabbits were cut open to cannulate the venae cavae at the jointure between the renal and iliac veins, establishing a constant flow of saline solution in order to clean the entire vascular circuit, avoiding the formation of clots that could adhere to the endothelium. The aorta was removed by a cut at the point where the two iliacs separate. Extracted vessels were denuded, everted and cut into six segments of 1 cm lenght. The endothelial layer was chemically removed with 20 U/mL α-chymotrypsin (37°C, overnight), to expose the subendothelium proteins. Vascular segments were stored in PBS at −20°C until used. Each experiment was performed with vessels from the same rabbit, to avoid inter-individual variations.

### Studies with flowing blood

Whole blood aliquots incubated with apixaban were recalcified with a CaCl_2_ solution, and immediately perfused through annular chambers exposing damaged vascular segments [21]. Perfusions studies were performed at a shear rate of 800 s^-1^, for 5 min. Perfused vessels were rinsed with 0,15 M PBS, fixed with 2.5% glutaraldehyde in 0.15 M PBS at 4°C for 24 h and histologically processed for further morphometric evaluation. Fibrin deposition and platelet interactions were evaluated by light microscopy in histological semi-thin cross-sections of the perfused vessels. A specifically developed software automatically classified and quantified platelet and fibrin coverage present in 20 randomly chosen microscopic fields in non-adjacent sections [22]. Platelets interactions with the exposed vascular surfaces were evaluated as a percentage of total surface of the vessel covered by platelet. Presence of larger platelet masses (aggregates of more than 5 μm in height) were also expressed as a percentage of the surface vessel. Similarly, the presence of fibrin in the same microscopic fields was also morphometrically quantified and expressed as a percentage of fibrin coverage deposited on the subendothelium.

### Thromboelastometry studies

Modifications on thromboelastometric parameters during clot formation of citrated blood samples previously incubated with apixaban (0, 10, 40 and 160 ng/mL) were investigated using the ROTEM Thromboelastometry Analyser (PentapharmGmbH, München, Germany) [21]. Clot formation was triggered by addition of ex-TEM^®^ reagent and recalcification with star-TEM^®^, both commercially available. The ex-TEM^®^ test measures changes on the extrinsic pathway of coagulation, fibrinogen and fibrin polymerization, and platelet function. Clots obtained in the ex-TEM^®^ are composed of platelets and fibrin. Clotting time (CT) and clot formation time (CFT) indicate the kinetics of clot formation and are expressed in seconds. Clot amplitude provides information about clot strength and stability, which is largely dependent on fibrinogen and platelets, and is expressed in millimetres. The CT is defined as the time when the forming clot reaches 2 mm; the CFT is the time when this clot reaches 20 mm; and the clot amplitude after 10 minutes (A10) is a measure of clot firmness at this time-point.

### A platelet-based model of thrombin generation

Contribution of platelets to local thrombin generation was investigated in an *in house* cell-based model of thrombin generation primed by platelets, using a modified fluorogenic assay (Technoclone GmBH, Austria). Our cell-based model consists of isolated platelets at 1x10^6^ platelets/μL in Hanks’ balanced salt solution, used as the source of anionic phospholipids and factors released from the granules after activation to enhance thrombin generation. Addition of 7.5% of citrated plasma ensured the presence of coagulation factors to support thrombin generation. This mix was incubated with the different concentrations of apixaban tested (0, 10, 40 and 160 ng/mL) for 30 min at 37°C. Parallel studies were performed using vehicle instead of platelets (S1 Supporting Information). Thrombin generation was initiated by addition of 1.1 pM tissue factor exposed on phospholipids micelles (Thromborel^®^S, Dade Behring, Marburg GmbH, Germany) and a fluorogenic substrate that also contains CaCl_2_ to favor the activation of coagulation mechanisms. Fluorescence generated was evaluated at a wavelength of 390 nm / 450 nm (excitation / emission) during 90 min (at intervals of 1 min) and fluorescence units analyzed with the Thermo Fluoroskan Ascent Software (Technoclone GmbH) as previously described [23]. Parameters assessed in our studies were lag phase (min), maximum thrombin peak (nM), time to achieve this peak (min) and total amount of thrombin generated (Area Under the Curve, A.U.C.).

### Statistics

Data are expressed as mean ± standard error of the mean (SEM). The SPSS statistical package 17.0.0 (SPSS, Inc, Chicago, IL) was used for all analyses. Statistical analysis was performed with raw data using the Student’s t-test for paired samples after Wilcoxon-Mann-Whitney test for Gaussian distribution. Minimal levels of statistical significance were established at p<0.05.

## Results

### Effects of apixaban on thrombus formed under flow conditions

**Fig 1:**
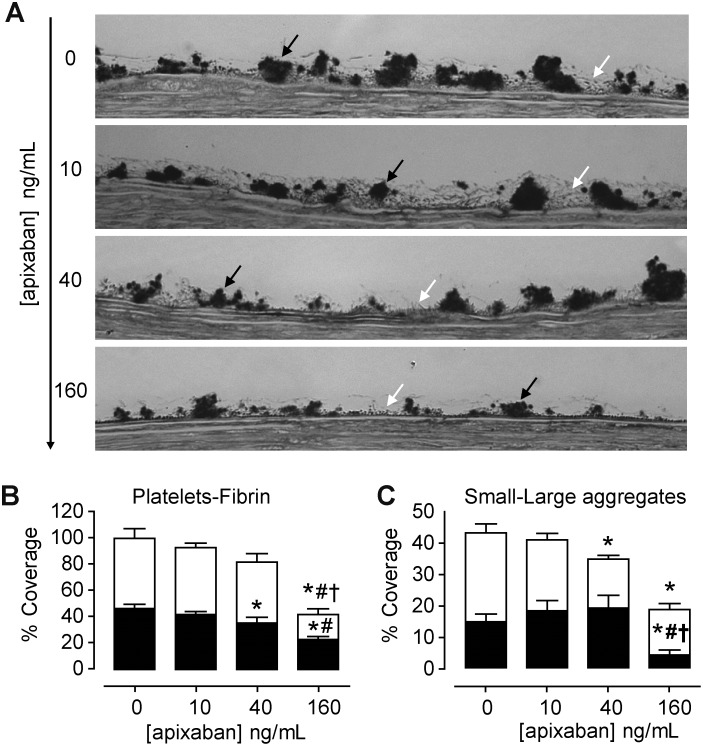
Effects of apixaban on platelet and fibrin interactions with damaged vessels exposed to flowing blood. At the highest concentration, apixaban significantly inhibited the incorporation of fibrin and platelets into the forming thrombi. Inhibitory effects on fibrin formation were not observed with apixaban at 40 or 10 ng/mL. Antiplatelet effects of apixaban were still evident at 40 ng/mL. (A) Representative micrographs showing platelets and fibrin interactions on cross-sections of the perfused vessels. Citrated blood previously incubated with different concentrations of apixaban (0, 10, 40 and 160 ng/mL) was recalcified and immediately perfused at a shear rate of 800 s^-1^, for 5 min. Black arrows point to platelet aggregates and white arrows indicate fibrin (Magnification x1000). (B) Bar diagrams represent results of morphometric evaluations in cross-sections of perfused vessels, showing percentage of surface covered by platelets aggregates (black) or fibrin (white). (C) Bar diagrams represent presence of small (< 5 microns height; black) and large (> 5 microns height; white) platelet aggregates. Results are expressed as Mean ± S.E.M (n = 5).* p<0.01 vs. baseline (no apixaban); # p<0.01 vs. apixaban 10 ng/mL and † p<0.01 vs. apixaban 40 ng/mL.

We assessed the effects of apixaban concentrations on platelet and fibrin components of thrombus formed in studies under flow conditions. As summarized in Fig 1A and 1B, apixaban at elevated concentrations (160 ng/mL) demonstrated a powerful inhibitory action on fibrin formation with a moderate reduction in platelet deposition with differences reaching values of statistical significance (p<0.01 vs. baseline values). Inhibitory effects on fibrin formation were not observed with the lower concentrations of apixaban tested (10 and 40 ng/mL) (Fig 1B).

A more detailed analysis of the characteristic of the platelet aggregates formed on the thrombogenic vessel surface revealed that apixaban interfered with the formation of larger platelet aggregates (≥ 5 microns in height) in a dose-dependent manner (Fig 1C). As shown in the bar diagrams, reduction in the proportion of larger aggregates resulted in a tendency to associate platelets into smaller platelet aggregates (< 5 microns in height). Apixaban at 160 ng/mL not only reduced the total surface covered by platelets, but also caused significant reductions in the formation of larger platelet aggregates (14.6 ± 1.9% vs. 28.4 ± 2.9% in controls; p<0.01). A similar tendency was observed with 40 ng/mL with an inferior but statistically significant reduction (p<0.05). The lower concentration of apixaban tested (10 ng/mL) did not significantly affect the formation or size of platelet aggregates. Results are summarized in Table 1.

**Table 1:** Modifications on percentages of platelet and fibrin coverage, by increasing concentrations of apixaban, in studies with flowing blood.

[Apixaban] ng/mL	Percentage of surface coverage (%)			
	Platelets	Fibrin	Large Agg.	Small Agg.
0	46.6 ± 3.5	53.8 ± 7.4	28.4 ± 2.9	14.6 ± 2.6
10	42.1 ± 2.5	51.2 ± 3.5	22.7 ± 2.0	18.1 ± 3.4
40	35.5 ± 4.8 *	46.9 ± 6.3	15.71 ± 1.3 *†	19.0 ± 4.3
160	21.0 ± 2.8 *†§	19.3 ± 4.4 *†§	14.6 ± 1.9*†	4.0 ± 1.8*†§

Large Agg.: Large platelet aggregates of > 5 microns in height

Small Agg.: Small platelet aggregates of <5 microns in height

Results are expressed as Mean ± SEM (n = 5)

* p<0.01 vs. apixaban 0 ng/mL

^**†**^ p<0.01 vs. apixaban 10 ng/mL

^**§**^ p<0.05 vs. apixaban 40 ng/mL

### Effects of apixaban on thromboelastometry parameters

**Fig 2:**
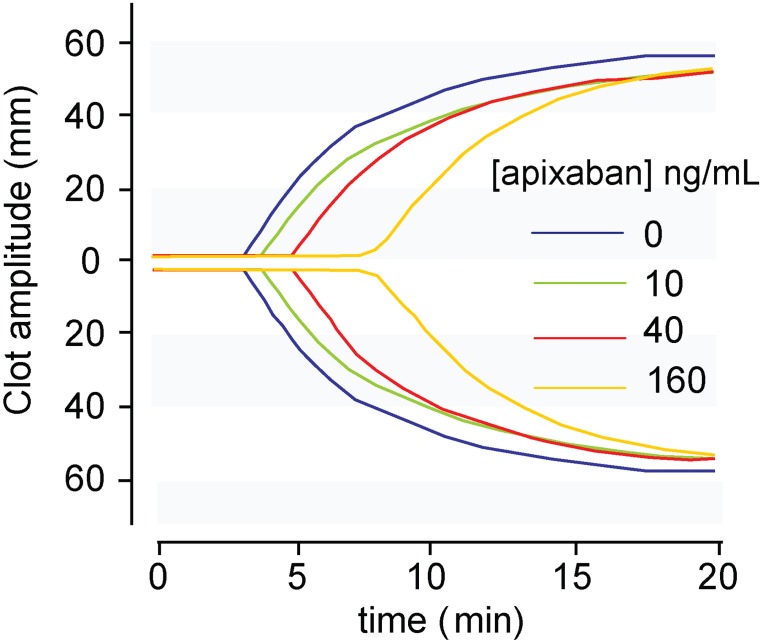
Modifications in thromboelastometry profiles of whole blood by apixaban at low and high concentrations. Assays were triggered by tissue factor-rich phospholipid micelles and calcium. Results reveal a dose-dependent action of apixaban (10, 40 and 160 ng/mL) on the kinetics of clot formation and clot firmness. Clotting times were progressively delayed by apixaban. Effects on clot firmness were less evident.

We also investigated modifications by apixaban in viscoelastic properties of forming clots. As shown in Fig 2, apixaban induced a dose-dependent alteration in the thromboelastometry parameters measured.

Exposure of human blood to 10, 40 or 160 ng/mL was followed by a progressive delay in the kinetics of clot formation, with prolongations of clotting related parameters (CT and CFT). These prolongations reached levels of statistical significance for concentrations of apixaban ≥40 ng/mL. Similar tendencies were observed for CFT. Evaluation of changes in the clot firmness showed a tendency to reduced clot firmness at 10 min (A10) with increasing concentrations of apixaban, but differences never reached levels of statistical significance. More detailed data is offered in Table 2.

**Table 2:** Modifications in thromboelastometry parameters by apixaban triggered by tissue factor rich phospholipid micelles and calcium.

apixaban (ng/mL)	CT (min)	CFT (min)	A10 (mm)
0	211.7 ± 13.3	95.7 ± 8.8	53.2 ± 2.3
10	247.8 ± 44.3	109.6 ± 9.1	52.4 ± 2.3
40	289.2 ± 48.6 #	129.2 ± 16.4	49.0 ± 2.9
160	367.7 ± 30.6 *#	141.0 ± 9.9 *#	47.7 ± 2.4

Apixaban delayed kinetics of clot formation, measured as clotting time (CT) and clot formation time (CFT); and reduced clot firmness after 10 minutes (A10). Results are expressed as Mean ± S.E.M (n = 5 to 6 for apix 0 and 160 ng/mL).

* p<0.05 vs. apixaban 0 ng/mL and

^#^ p<0.05 vs. apixaban 10 ng/mL

### Impact of apixaban on thrombin generation primed by platelets

**Fig 3:**
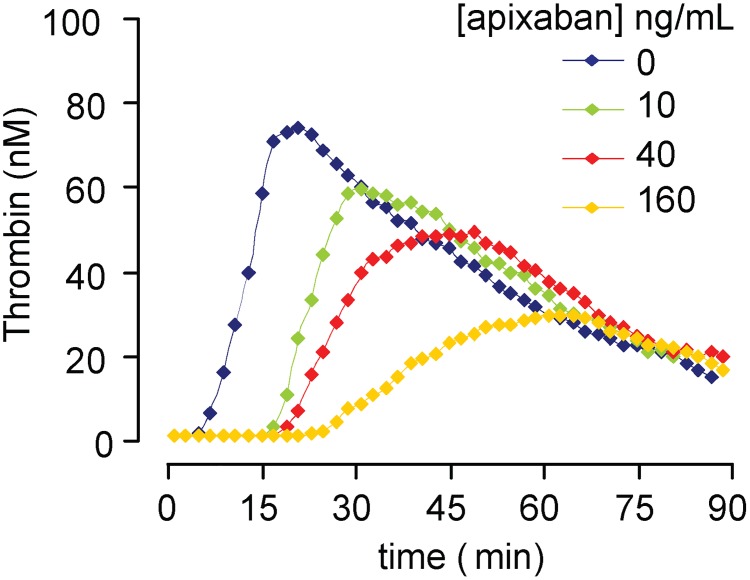
Impact of apixaban in a platelet-primed model of thrombin generation. Representative thrombin generation profiles showing the impact of different doses of apixaban in the contribution of platelets to local thrombin generation primed by platelets, triggered by tissue factor rich phospholipids-micelles and calcium. Apixaban caused dose-dependent prolongations in the initiation of thrombin generation with a parallel reduction in the thrombin peak and the total amount of thrombin generated. This assay detected alterations in the kinetics of thrombin generation and total amount of thrombin generated, even with the lowest concentrations of apixaban tested (10 and 40 ng/mL).

Contribution of platelets to local thrombin generation was investigated in a modified cell-based model of thrombin generation primed by platelets. Representative thrombin generation profiles are shown in Fig 3. Normal values for the lag phase in experiments in the absence of apixaban were 7.0 ± 0.8 min, with a maximum peak of thrombin equivalent to 79.1 ± 4.9 nM, reached at 21.8 ± 3.1 min.

Apixaban showed a clear dose effect relationship in the kinetics of thrombin generation, with progressive delays in both the time to start thrombin generation (lag time), which was doubled and maintained with all the apixaban concentrations tested (lag times ranging from 13 min to 13.4 min), and the time to reach the maximum peak of thrombin, which was prolonged in a dose-dependent manner (37.1 ± 1.9 min, 57.8 ± 8.8 min and 72.5 ± 6.2 min, respectively with apixaban 10, 40 and 160 ng/mL). These reductions reached statistical significance with all the apixaban concentrations investigated vs. control experiments (p<0.01). Moreover, the maximum peak of thrombin generated was reduced proportionally to the dose of apixaban (60.9 ± 1.0 nM, 55.7 ± 3.5 nM and 36.7 ± 3.1nM, respectively with apixaban 10, 40 and 160 ng/mL), reaching statistically significant inhibitions with all the concentrations of apixaban when compared to control studies performed in the absence of this anticoagulant (p<0.01). Thrombin peak and area under the curve (A.U.C.) reached with 160 ng/mL of apixaban were significantly reduced with the respective results observed for concentrations of 10 or 40 ng/mL (p<0.01). For more detailed results, including those in experiments with vehicle, refer to Table 3 and S1 Table, respectively.

**Table 3:** Modifications on thrombin generation parameters induced by increasing concentrations of apixaban in platelet-enriched samples.

[Apixaban] ng/mL	Lag Phase (min)	Thrombin Peak		Area Under Curve A.U.C.
		Time (min)	Peak (nM)	
0	7.0 ± 0.8	21.8 ± 3.1	79.1 ± 4.9	3156.8 ± 96.5
10	13.0 ± 0.5 *	37.1 ± 1.9 *	60.9 ± 1.0 *	2664.2 ± 87.2 *
40	13.4 ± 1.4 *	57.8 ± 8.8 *	55.7 ± 3.5 *	2447.3 ± 178.0 *
160	13.4 ± 0.9 *	72.5 ± 6.2 *†	36.7 ± 3.1 *†§	1421.8 ± 234.1 *†§

Results are expressed as Mean ± SEM (n = 5)

* p<0.01 vs. apixaban 0 ng/mL

^**†**^ p<0.01 vs. apixaban 10 ng/mL

^**§**^ p<0.05 vs. apixaban 40 ng/mL

## Discussion

Apixaban showed variable antithrombotic actions in the different experimental approaches investigated. In flow studies in a thrombosis model with blood circulating at arterial shear rates, apixaban concentrations equivalent to the C_max_ achieved in patients receiving treatment for the prophylaxis of thrombotic complications, significantly reduced fibrin generation and platelet aggregate formation. These effects on platelets and fibrin formation were not observed with the lower concentrations of apixaban tested. In contrast with the previous observations, concentrations 4 to 16 times below the usual antithrombotic C_max_ did still show significant inhibitory action in the contribution of platelets to thrombin generation in studies under more static conditions. A combination of studies with flowing blood and thrombin generation in a platelet-rich environment could be useful to differentiate concentrations of anticoagulants that inhibit platelet-mediated thrombin formation from those that deeply impair fibrin formation necessary for consolidation of hemostasis.

Our present studies demonstrate a differential impact of high vs. low concentrations of apixaban on platelet and coagulation components of thrombus formation in studies under flow conditions. Previous studies from our group demonstrated that apixaban at a concentration compatible with the C_max_ reached in patients subjected to treatment for the prophylaxis of thrombotic complications [18,19,21,24], dramatically reduce fibrin incorporation into forming thrombi in a similar thrombosis model with human blood circulated at moderated shear rates. Our present results with apixaban at 160 ng/mL indicate that, in addition to its powerful inhibitory action on fibrin formation, apixaban interfered with the formation of large platelet aggregates causing statistically significant reductions in their size. Interestingly, the lower concentrations of apixaban (40 or 10 ng/mL) did not cause significant quantitative modifications in fibrin deposition, though they still showed some antiplatelet effects under dynamic flow conditions. Therefore our results support the concept that low concentrations of apixaban inhibit platelet-mediated thrombin generation that could facilitate the formation of platelet-rich occlusive thrombi.

Blood flow conditions play a critical role in the pathophysiology of venous and arterial thrombotic complications [25]. Venous and intracardiac thrombi are generated under relatively low-shear environments that favor the activation of the coagulation mechanisms, thus generating fibrin-rich thrombi. In contrast, platelet adhesion and aggregation play a predominant role at the elevated shear rates that occur in arterial regions resulting in rapidly developing thrombi that contribute to the ischemic complications in ACS [26,27]. Flow conditions are subjected to extreme variations during the formation of an occlusive thrombi shifting from extremely elevated shear rates at the apex of a growing thrombi, to reduced shears related to deceleration of blood flow at the base of the thrombi, or to almost stagnant conditions in the distal area were the thrombi is being formed [28,29]. Under these circumstances, platelets circulating close to the vessel wall interact with aggregated platelets already deposited on a damaged atherosclerotic plaque, being further activated by thrombin locally generated, and favoring thrombus propagation [9]. Our experimental design wanted to reproduce this scenario by evaluating the contribution of platelets to thrombin generation in a modified version of the cell-based model of coagulation in the presence of elevated number of platelets, tissue factor and diluted plasma [30]. Our results in this thrombin generation assay primed by platelets differ from those in the studies with flowing blood. While the inhibition of thrombin generation followed a dose-effect relationship with the concentration of apixaban in the thrombin generation assay, the inhibition of fibrin formation and platelet deposition was only observed with the most elevated concentration of apixaban in studies with circulating blood. These findings confirm that concentrations of apixaban required to inhibit fibrin formation under flow conditions are much higher than those necessary to significantly inhibit thrombin generation in a platelet rich environment.

Thrombin locally generated plays a critical role in the composition and structure of hemostatic and occlusive clots through the activation of platelets and generation of a fibrin net that will consolidate growing thrombi. In hemostatic clots, fibrin stabilizes the initial platelet-rich plug to control bleeding through an injured vessel [31]. Fibrin formation also contributes to the formation of pathologic clots under elevated arterial shear rates leading to an increase in platelet presence and a low porosity fibrin packing that limits the diffusion of thrombin [16]. At the early stages of formation of pathologic arterial clots thrombin generated is rapidly removed by the bloodstream leaving only thrombin generated at the core to activate and recruit additional platelets [32]. As the thrombus becomes more occlusive, thrombin generated incorporates more fibrin and platelets, making it more resistant and reducing its porosity [28,29]. Inhibition of local thrombin generated at the level of a platelet thrombus consolidating on a ruptured plaque, should prevent the feedback of thrombin generated promoting the activation of further circulating platelets and the association of fibrin and VWF to the forming thrombus. Our data indicate that the lower concentrations of apixaban tested in our studies may interfere with this feedback mechanism, and still preserve the formation of hemostatic fibrin clots.

In our studies, apixaban delayed kinetic thromboelastometry parameters, but seemed to have minimal impact on the structural properties of the formed clots. The prolongation of clotting times with the increasing doses of apixaban paralleled the reductions measured in thrombin generation kinetics. In contrast, apixaban as other DOACs did not modify in a significant way the maximum clot firmness of clots formed in the ROTEM system [21,33]. Our thromboelastometry studies reflect the antithrombotic action of apixaban in hemostatic and venous clots as a prolongation of clotting times related to a reduced and delayed generation of thrombin. However, the composition of clots generated under the low shear conditions produced in thromboelastometry would be completely different from the platelet-rich occlusive thrombi generated in ruptured atherosclerotic plaques. It is likely that thromboelastometry results do not bring additional information on the structure and clot resistance of clots forming ant elevated shear rates.

Results of our present studies may have clinical implications. Combined inhibition of fibrin formation and platelet aggregation caused by DOACs may interfere with hemostasis and result in increased bleeding. Actually, an enhancement of the antithrombotic action of rivaroxaban with single or dual antiplatelet therapy has been demonstrated in a porcine model of stent thrombosis [34]. The APPRAISE-2 trial investigated the concomitant beneficial effect of apixaban and standard antiplatelet therapy in ACS; however, the dose of 5 mg twice daily (BID), equivalent to the full anticoagulant dose recommended for SPAF revealed unsatisfactory outcomes [35]. This clinical trial was prematurely terminated due to an increase in major bleeding events with apixaban in the absence of a counterbalancing reduction in recurrent ischemic events. Interestingly, in the phase III study ATLAS ACS 2 TIMI 51 patients with a recent ACS were randomized to rivaroxaban 2.5 mg BID or 5 mg BID or placebo, in addition to standard antiplatelet therapy [36]. In the mentioned study, rivaroxaban -at doses one fourth to one half of those indicated for the prevention or treatment of venous or AF-related thrombosis- reduced the risk of the composite end-point of death from cardiovascular causes, myocardial infarction, or stroke in patient with recent acute coronary syndromes. Moreover, evidence from a recent clinical trial confirms the efficacy and safety of the 2.5 mg rivaroxaban dose (2.5 mg BID in patients with atrial fibrillation undergoing intracoronary stenting, receiving dual antiplatelet therapy [37]

It is quite possible that the selection of doses of apixaban for the prevention on thrombotic complications in arterial territories was based on those established for other antithrombotic indications and did not consider the antihemostatic action of the elevated doses of apixaban at elevated shear rates. Our data indicate that lower concentrations of apixaban may interfere with thrombin generation in a platelet rich environment, but still preserve the contribution of fibrin to the preservation of its hemostatic function.

## Conclusion

In the present study, apixaban at 10, 40 and 160 ng/mL differently inhibited local thrombin generation, altered coagulation parameters during the clot formation, and reduced platelet and fibrin deposition on damaged vessels in studies with circulating blood. Our data indicate that apixaban at doses one fifth to one half of that indicated for the prevention or AF-related thrombosis, could be adequate to reduce thrombin generation, the formation of large platelet aggregates and still allow fibrin formation, thus preserving its contribution to hemostasis. Studies in modified thrombin generation assays primed by platelets could be useful to investigate the antithrombotic action of DOACs in the prevention of atherothrombotic complications in arterial territories. Moreover, experimental studies under flow conditions combined with modified thrombin generation assays would facilitate the selection of DOAC concentration that prevents thrombin-related recruitment of platelets in pathological arterial thrombi, reducing the risk of interfering with fibrin formation necessary for the consolidation of hemostasis.

## Supporting information

S1 Supporting information Thrombin generation in a cell-based model primed by platelets vs. vehicle. (DOCX)

S1 Table Parameters of thrombin generation primed by platelets *vs*. vehicle. (DOC)
