# Supplementary material for: Differential inhibitory action of apixaban on platelet and fibrin components of forming thrombi: Studies with circulating blood and in a platelet-based model of thrombin generation
Source: PLoS One. 2017 Feb 13;12(2):e0171486. doi: 10.1371/journal.pone.0171486 (PMC5305231; doi:10.1371/journal.pone.0171486)
Supplement: S1 Supporting information — (DOCX) [file pone.0171486.s001.docx]

**S1 Supporting Information. Thrombin generation in a cell-based model primed by platelets vs. vehicle.** To emphasize the contribution of platelets to thrombin generating in the arterial vessels we adapted a fluorimetric assay of thrombin generation and established the optimal conditions: platelets (1x10^6^/µL), which contribute to the process with anionic phospholipids and released factors from the granules after activation; and 7.5% plasma, which provides coagulation factors enough to initiate and maintain the thrombin generation. These samples were incubated for 30 min with apixaban. The reaction of thrombin generation assay was initiated with a preparation of tissue factor (1.1 pM)$\mu$, calcium and fluorogenic substrate (Technothrombin). Generated fluorescence was recorded for 90 min (λ ex/em: 390/450 nm). Concentrations of platelet and plasma previously assessed showed the most reliable results to study inhibition of thrombin generation by apixaban in the presence of an elevated concentration of platelets to (1x10^6^/µL).Thrombin generation was extremely reduced in experiments with 7.5% of plasma, in the absence of platelets. However, presence of platelets restored and potentiated thrombin generation in this setting. Apixaban showed a clear dose-dependent inhibitory action on thrombin generation primed by platelets, even with the lowest concentration tested (10 ng/mL), approximately 16-fold below the therapeutic C_max_. Parallel determinations using vehicle (Hanks’s balanced salt solution) instead of platelets demonstrated critical delays by apixaban in the dynamic parameters of thrombin generation far exceeding those in the presence of platelets, with minimal amounts of thrombin generated during the assay ranging between 0 nM to 5.78 nM (average 3.8 ± 1.0 nM). For more detail please refer to S1 Table.
