# Supplementary material for: Differential inhibitory action of apixaban on platelet and fibrin components of forming thrombi: Studies with circulating blood and in a platelet-based model of thrombin generation
Source: PLoS One. 2017 Feb 13;12(2):e0171486. doi: 10.1371/journal.pone.0171486 (PMC5305231; doi:10.1371/journal.pone.0171486)
Supplement: S1 Table — (DOC) [file pone.0171486.s002.doc]

**S1 Table**. **Parameters of thrombin generation primed by platelets *vs.* vehicle.**

| [Apixaban] ng/mL | platelets + 7.5 % PPP | | | | vehicle + 7.5 % PPP |  |
| --- | --- | --- | --- | --- | --- | --- |
| Lag Phase (min) | | |  |  |  | |
| 0 | 7.0 ± 0.8 | | | | 16.2 ± 2.8 |  |
| 10 | 13.3 ± 0.5 ***** | | | | 17.8 ± 1.7 |  |
| 40 | 13.4 ± 1.4 ***** | | | | 23.2 ± 2.0 # |  |
| 160 | 13.4 ± 0.9 ***** | | | | 17.6 ± 2.4 # |  |
| Thrombin Time (min) | |  | | | | |
| 0 | 21.8 ± 3.1 | | | | 42.4 ± 18.1 |  |
| 10 | 37.1 ± 1.9 ***** | | | | 44.4 ± 19.9 |  |
| 40 | 57.8 ± 8.8 ***** | | | | 54.4 ± 16.5 |  |
| 160 | 72.5 ± 6.2 ***†** | | | | 31.2 ± 13.9 |  |
| Thrombin Peak (nM) | |  | | |  | |
| 0 | 79.1 ± 4.9 | | | | 3.8 ± 1.0 # |  |
| 10 | 60.9 ± 1.0 ***** | | | | 5.6 ± 2.5 # |  |
| 40 | 55.7 ± 3.5 ***** | | | | 2.6 ± 1.0 # |  |
| 160 | 36.7 ± 3.1 ***†§** | | | | 1.9 ± 0.6 # |  |
| Area Under the Curve (A.U.C.) | | | | | |  |
| 0 | 3156.8 ± 96.5 | | | | 106.8 ± 51.4 **#** |  |
| 10 | 2664.2 ± 87.2 ***** | | | | 103.6 ± 46.1 **#** |  |
| 40 | 2447.3 ± 178.0 ***** | | | | 26.1 ± 11.7 **#** |  |
| 160 | 1421.8 ± 234.1 ***†§** | | | | 3.5 ± 2.1 **#** |  |

Rows compare measurements between samples with platelets and vehicle for the same concentration of apixaban. Results expressed as Mean ± SEM (n = 5).

***** p<0.01 vs. the corresponding control (platelets / vehicle) with apixaban at 0 ng/mL

**†** p<0.01 vs. apixaban 10 ng/mL

**§** p<0.05 vs. apixaban 40 ng/mL

**#** p<0.05 vs. the same apixaban concentration in platelets + 7.5 % PPP
